# Clinical Manifestations and Molecular Biology of One Case of Carney Complex: A Case Report

**Published:** 2018-04

**Authors:** Mengxue YANG, Biao LONG, Jie XU, Jie YU, Xianwen LI, Fanhao YE, Bo YANG, Yulan LIAO, Sicheng LI, Ya LI, Xue ZHOU

**Affiliations:** 1. Dept. of Endocrinology, Affiliated Hospital of Zunyi Medical University, Zunyi, China; 2. Dept. of Pediatrics, Qiandongnan People’s Hospital, Kaili, Guizhou, China; 3. School of Public Health, Zunyi Medical University, Zunyi, China

**Keywords:** Primary pigmented nodular adrenocortical disease, Carney complex, Point mutation, *PRKAR1A* gene

## Abstract

Carney complex (CNC) is a rare genetic disease. Here, we report a case of CNC and explore clinical manifestations and gene mutation studies of CNC. A male patient with CNC at the age of 16 yr was admitted to Affiliated Hospital of Zunyi Medical University in July, 2015. Although the patient had typical signs of Cushing’s syndrome, he also presented with certain rare signs of Cushing’s syndrome, such as “freckle-like” scattered spots of pigmentation on the face and around the lips. In addition, concomitant severe osteoporosis led to flattened vertebrae and the compression of corresponding levels of the spinal cord. Radiographic findings revealed adrenal nodular hyperplasia. Based on sequencing, 2 novel heterozygous mutations of the *PRKAR1A* gene were found. CNC was eventually diagnosed via pathologic biopsy. After 1 year of follow-up, the patient exhibited weight loss, relief of low back pain, normal blood biochemical indicators and cortisol levels at the lower limit of the normal range.

## Introduction

Carney complex (CNC) is a rare genetic disease first described by J. Aidan Carney as a complex of myxomas, spotty pigmentation and endocrine overactivity (1). The *PRKAR1A* gene is involved in the pathogenesis of CNC, which exhibits dominant inheritance within families. *PRKAR1A* is a type of tumor suppressor gene, and mutations in *PRKAR1A* may relate to the onset of endocrine tumors (2). Among various symptoms of CNC, primary pigmented nodular adrenocortical disease (PPNAD) is observed in 25% of cases. PPNAD, which is the only inherited form of Cushing’s syndrome, is the most common endocrine adenomatous lesion associated with CNC (2, 3). The incidence of PPNAD in cases of CNC varies from 20% to 62.5% across different reports (4–6).

The main clinical manifestations of CNC include hypercortisolism, cardiac myxomas, thyroid neoplasms and skin pigmentation among others. Clinical manifestations of CNC are complex and diverse, with significant individual differences, and CNC lacks specific manifestations and imaging characteristics; as a result, this disease can be quite easily misdiagnosed or missed in clinical practice, a phenomenon that affects the treatment and prognosis of patients with CNC. Therefore, special attention should be devoted to the clinical diagnosis and treatment of CNC. Here we report a case of CNC diagnosed in Jul 2015.

## Case report

A 16-yr-old male patient was admitted to the Affiliated Hospital of Zunyi Medical University (Zunyi, China) in July 2015, due to weight gain over 3 yr, purple striae on the skin for 1 year and low back pain for 2 months. He began to gradually gain weight 3 yr ago for no obvious reason. His low back pain was exacerbated because he accidentally fell 2 months ago, and this pain had persisted until 1 day before admission. Since the onset of his disease, the patient felt that he was shorter and had a worse mentality than his peers. He underwent tumor resection in the upper right arm three years ago at a local hospital; however, no pathological examination was conducted at that time. His father and mother were 168 cm and 158 cm, respectively. There was no relevant family history.

**Fig. 1:**
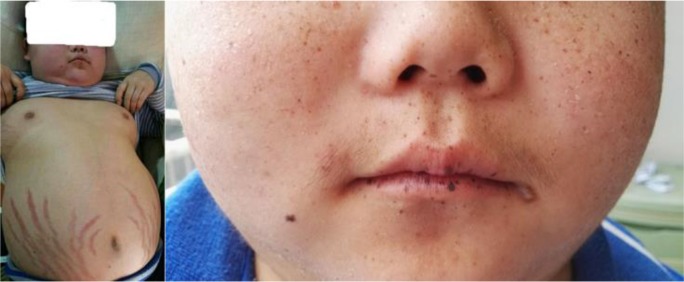
Typical signs of Cushing’s syndrome (left, A) and scattered spotty pigmentation on the face (right, B)

Physical examination revealed a body temperature of 36.4 °C, a pulse rate of 80 beats/min, a respiratory rate of 18 breaths/min and a blood pressure of 112/72 mmHg. His body weight was 60 kg, and 120 cm; thus, his body mass index was 41.67 kg/m^2^. His upper body was 76 cm, his lower body was 72 cm, and his fingertip distance was 147 cm. He exhibited central obesity, a full-moon face, a buffalo hump, increased hair and scattered facial and perioral spots with pigmentation (Fig. 1A). Broad purple striae were found on the skin of the left armpit and the lower abdomen (Fig. 1B).

The patient’s external genitalia were puerile, and the left testis was relatively small. The following findings were obtained from laboratory tests: blood uric acid of 537 μmol/L, 24 h uric acid of 2077 mol, fasting C-peptide of 2060 pmol/L, fasting insulin of 16 μIU/mL, 2 h postprandial C-peptide of 3060 pmol/L and 2 h postprandial insulin of 37.2 μIU/mL. Adrenocorticotropic hormone (ACTH) and cortisol exhibited circadian rhythm. The cortisol level determined using a low-dose (0.75 mg tid) dexamethasone resistance test was 462.9 nmol/L and could not be inhibited even during a high-dose dexamethasone suppression test (2 mg q6h); the patient’s cortisol level was abnormally elevated (Table 1).

**Table 1:** High-dose dexamethasone suppression test

***Test item***	***8:00***	***16:00***	***24:00***
ACTH (pg/mL)	<1.0	1.54	1.54
Cortisol (pre-suppression) (nmol/L)	788.6	573.6	289.0
Cortisol (post-suppression) (nmol/L)	906.5		

**Fig. 2:**
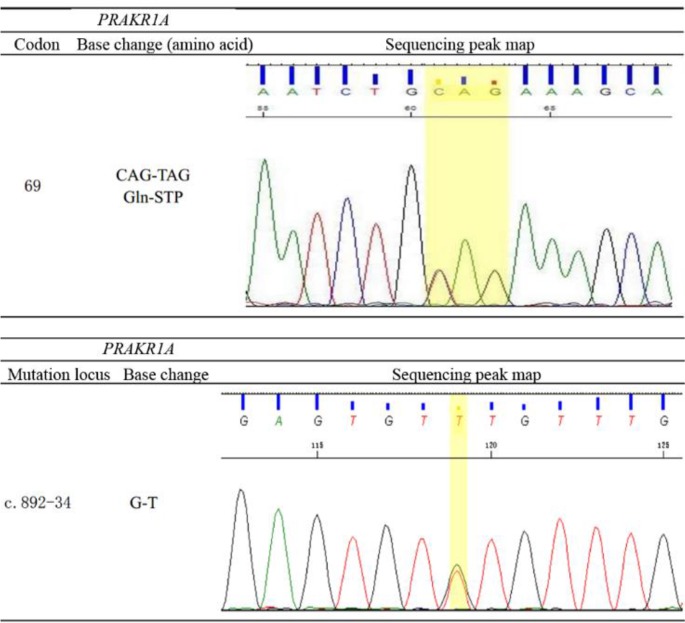
Results of the *PRAKR1A* gene test

The patient’s insulin-like epidermal growth factor and growth hormone (GH) levels were 10.1 mIU/L and 0.1 mIU/L, respectively. An insulin-based hypoglycemia-stimulating GH test was positive. Color Doppler of the scrotum suggested bilateral testicular microlithiasis. Computed tomography (CT) of the adrenal glands revealed slight bilateral thickening. The bone mineral density test yielded a lower result than is typically obtained for the patient’s peers. The results from a left wrist plain film were consistent with bone age imaging findings for 14-yr-old children, with epiphyses not closed. Thoracic vertebral magnetic resonance imaging (MRI) indicated that the 5^th^–7^th^ thoracic vertebrae had become flattened and that corresponding levels of the spinal cord were compressed. Plain scanning of lumbar vertebrae revealed findings indicative of a compression fracture, such as multiple flattened thoracolumbar vertebrae and signal abnormalities. This scanning also revealed atrophic degeneration in the 5^th^–12^th^ thoracic spinal cord segments. Normal findings were obtained for a sex hormone examination; a 17-hydroxyprogesterone test; a thyroid function test; color Doppler ultrasound of the thyroid and heart; and MRI of cervical vertebrae, the head and the pituitary. The *PRAKR1A* gene test (Fig. 2) (Chinese National Human Genome Center, Beijing, Oct 20, 2015) revealed mutations at the 34^th^ base of the intron before coding exon 9 and the 69^th^ codon of coding exon 3 of *PRKAR1A*.

On Feb 19, 2016, the patient underwent posterior laparoscopic right adrenalectomy under general anesthesia; posterior laparoscopic partial left adrenalectomy was performed under general anesthesia on Mar 1, 2016. Postoperative pathological examination suggested multinodular hyper-plasia in the right adrenal cortex and left adrenal morphology consistent with cortical nodular hyperplasia. After discharge, the patient was prescribed a regimen involving the daily administration of a 5 mg prednisone tablet. Re-examination revealed that the patient’s cortisol levels were 56.00 ng/mL, 134.00 ng/mL and 74.00 ng/mL at 8:00, 16:00, and 24:00, respectively. During 1 year of follow-up, the patient’s body weight significantly decreased relative to pre-treatment levels, his low back pain was alleviated, blood biochemical parameters returned to normal, and cortisol levels were within the lower part of the normal range.

The current report was approved by the Ethics Committee of the Affiliated Hospital of Zunyi Medical University, and informed content was obtained from the guardian of the patient.

## Discussion

This patient had typical clinical manifestations of Cushing’s syndrome. The detected cortisol and ACTH levels, adrenal imaging findings and high- and low-dose dexamethasone suppression results, the patient was diagnosed with non-ACTH-dependent Cushing’s syndrome; the postoperative pathological diagnosis was PPNAD. PPNAD is one of various clinical manifestations of CNC, a rare autosomal dominant genetic syndrome characterized by pigmentation of the skin and mucous membranes; myxomas of the heart, skin and other body parts; and multiple endocrine tumors. However, since the patient exhibited the specific manifestation of melanin spots on his lips and mucosa, he could easily have been misdiagnosed with “freckles.”

*PRKARlA* is a tumor suppressor gene that encodes the protein kinase A (PKA) Iα regulatory subunit. Normally, this protein combines with the C catalytic subunit of PKA to form a stable tetramer (containing 2 Iα regulatory subunits and 2 C catalytic subunits) (7–10). Once PKA is activated by upstream signals, the α subunit will bind to cAMP and dissociate from the catalytic subunit, allowing the C subunit to play a catalytic role that activates the downstream CREB signaling system to promote DNA replication, cell growth, and proliferation. The two α subunits are encoded by two individual chromosomes. Therefore, when a relevant gene on one of these chromosomes is mutated, causing abnormally reduced protein synthesis, PKA will lack an α subunit and will not be able to maintain a stable tetramer structure, leading to less inhibition of PKA; downstream signals will be continuously activated, ultimately causing abnormal cell growth and proliferation (1, 10, 11).

Since CNC was first recognized, the majority of the 400 reported cases of CNC have exhibited familial aggregation (2). Molecular genetic studies have revealed that CNC is dominantly inherited within a family and linked to the 17q22-24 region. Mutations in the cAMP-dependent PKA regulatory subunit Iα (*PRKAR1A*) gene have been confirmed to be responsible for 45% of familial and sporadic cases of CNC. Among symptoms of CNC, PPNAD is observed in 25% of CNC cases. As the only inherited form of Cushing’s syndrome, PPNAD is associated with the most common endocrine adenomatous lesion of CNC (2). PPNAD often manifests at a younger age than Cushing’s syndrome induced by any other cause. CNC has the following characteristics: it is common in young people (10–20 yr); it typically presents with small nodular hyperplasia; patients exhibit low or undetectable blood ACTH levels; high-dose dexamethasone cannot reduce blood cortisol levels, which undergo repeated and unexplained abnormal increases; patients are positive for adrenal excitatory immunoglobulin; and onset is correlated with CNC-related genetic mutation and may be associated with mesenchymal tumors (particularly atrial myxomas), skin pigmentation and peripheral nerve damage (12). In laboratory diagnoses of CNC, a dexamethasone suppression test is preferred; during this test, PPNAD patients often exhibit abnormally elevated cortisol levels (13).

The patient described here was a 16-yr-old boy who suffered from growth stagnation since his disease has a prominent effect on patients’ growth and development. The patient also presented with typical manifestations of Cushing’s syndrome, including a full-moon face, a buffalo hump, purple skin striae, other signs of central obesity, and severe osteoporosis. Neither high- nor low-dose dexamethasone suppression effectively lowered the patient’s cortisol levels.

The high-dose dexamethasone suppression test produced not merely unsuppressed but abnormally elevated cortisol levels. This abnormal increase may be associated with the abnormally increased expression of cortical hormone receptors within adrenal nodules, which leads to increased targets of dexamethasone in the adrenal cortex (14). The c.892-34 locus of the patient’s *PRKAR1A* gene was mutated from G to T to yield a heterozygous GT genotype; the 34th base in the intron preceding coding exon 9 was mutated from G to T. This mutation may produce a mutation in the protein encoded by *PRKAR1A* and was the cause of the described case of CNC. Since this site is the mRNA splicing site, mutations in a single base may lead to posttranslational splicing errors and the premature termination of mRNA transcription. If the transcription levels remain normal, the aforementioned mutation causes the mutation of a methionine into an argi-nine. The disulfide bond provided by methionine plays an important role in maintaining the high-level structure of the protein. Thus, a mutation at the aforementioned site will preclude the formation of this disulfide bond; as a result, the protein structure will be destroyed, and the protein’s activity will be lost. Therefore, we deduce that regardless of whether a mutation at this locus results in splicing errors or amino acid substitution, the stability of the PKA tetramer will be markedly affected, resulting in the occurrence of endocrine adenomas. The point mutations discussed above have not been detected at the RNA level in diseased tissue (3). At present, surgery is the first-choice treatment for CNC; in particular, bilateral adrenalectomy is the typical approach. The patient described here underwent bilateral adrenalectomy. Postoperative pathological findings revealed multinodular hyperplasia in the right adrenal cortex and left adrenal gland morphology consistent with nodular hyperplasia. Reexamination indicated that the patient’s cortisol level was decreased, his low back pain was relieved, his skin purple striae were reduced and his symptoms were improved. However, two weeks after surgery, the patient began to suffer from diarrhea, nausea and other gastrointestinal symptoms, and the relevant hormone levels exhibited a downward trend. These conditions improved after hormonal treatment with hydrocortisone.

After discharge, the patient was asked to continue replacement therapy with oral prednisone acetate tablets. Further follow-up is needed to monitor hormone levels and changes in the disease course.

## Conclusion

The current case report led to the following conclusions. When a patient between 10 yr and 20 yr presents with typical signs of Cushing’s syndrome, great caution should be given to Carney complex induced by adrenal nodular hyperplasia. In laboratory diagnosis, dexamethasone suppression test is the preferred method. Gene detection plays a key role in diagnosis, but pathological biopsy is necessary for confirmation. Surgery is the treatment of choice for Carney complex.

## Ethical considerations

Ethical issues (Including plagiarism, informed consent, misconduct, data fabrication and/or falsification, double publication and/or submission, redundancy, etc.) have been completely observed by the authors.
